# Supplementary material for: Supracellular contractility in Xenopus embryo epithelia regulated by extracellular ATP and the purinergic receptor P2Y2
Source: J Cell Sci. 2025 Oct 2;138(18):jcs263877. doi: 10.1242/jcs.263877 (PMC12516192; doi:10.1242/jcs.263877)
Supplement: Supplementary information [file joces-138-263877-s1.pdf]

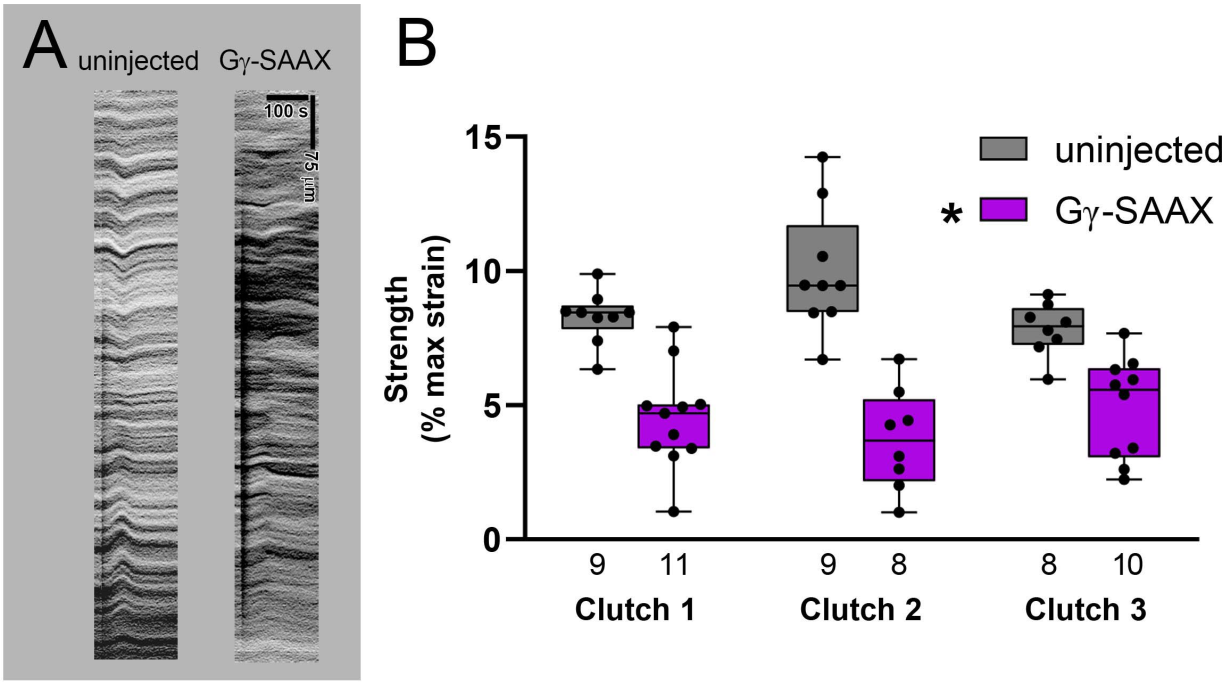

Fig. S1. Gγ-SaaX proteins reduce contractility. A) Kymographs of Xenopus ectoderm expressing Gγ-SaaX dominant negative constucts shows reduced contractility when perfused with 40 nl of 40 μM ATP. B) The strength of contractility is measured as in Fig. 2B. The number of embryos in each set is indicated below the data column. Statistics in (B) indicates significance of the Strength of Contraction (*, $p < 0.05$).

Table S1. Testing compounds for ability to induce epithelial contraction.

	Candidate Factors	# of embryos	%[1]	Contraction Magnitude[2]
**Controls**	Lysate (2 embryos lysed per 100 µl 1/3X MBS)	49	100	+ +
	1/3X MBS	6	0	-
	20% Ink (Higgins Fountain Pen India Ink) in 1/3X MBS	6	0	-
**Salts**	Potassium chloride (KCl, 1M)	6	0	-
	Sodium chloride (NaCl, 1M)	6	0	-
	Calcium chloride ($CaCl_2$, 1M)	6	0	-
	Magnesium chloride ($MgCl_2$, 1M)	5	0	-
**Bioactive Compounds**	Sodium glutamate ($C_5H_8NNaO_4$, 1M)	3	0	-
	Acetylcholine (3.1 mM)	3	0	-
**Treatments**	Lysate - heat treated (65 °C, 30 minutes)	9	100	++
	Lysate - shrimp alkaline phosphatase treated (SAP, 1U/ 10 µl)	5	0	-

[1]Incidence of contraction may be any magnitude (++ or +) in response to perfusion. [2]Contraction magnitude is qualitatively assessed for trial perfusions of embryos with an observable contraction. Contraction magnitude is categorized with respect to 400 µM ATP or Lysate: (++) contraction is equal or stronger, (+) contraction is weaker, (-) contraction not observed.

Journal of Cell Science • Supplementary information

Table S2. Testing inhibitors for ability to block epithelial contraction by lysate or ATP.

	Candidate Inhibitors	Perfused with	# of embryos	%[1]	Contraction Magnitude[2]
**Embryos incubated in compounds for 2 hours**	Lanthanum chloride (4 mM; L-type Calcium channels; (Lettvin et al., 1964))	Lysate	20	100	++
	Pyridoxalphosphate-6-azophenyl-2',4'-disulphonic acid (PPADS 100 μM; P2X/P2Y; (Lambrecht et al., 1992))	ATP (400 μM)	6	100	++
		ATP (40 μM)	8	100	++
		ATP (4 μM)	4	100	++
		ATP (0.4 μM)	8	0	-

[1]Incidence of contraction may be any magnitude (++ or +) in response to perfusion. [2]Contraction magnitude is qualitatively assessed for trial perfusions of embryos by comparison with an observable contraction. Contraction magnitude is categorized with respect to 400 μM ATP or Lysate: (++) contraction is equal or stronger, (+) contraction is weaker, (-) contraction not observed.

Table S3. PCR Expression Profile of P2YR.

	Primer 1	Primer 2	St 0	St 8	St 10	St 10 ecto
P2Y1.L/S	CTCAAAGCACTTTGCTGGCAAGTG	GCTTGTGTCCCCATTCTGCTTG	-	-	-	-
P2Y2.L	CGTCCTCCTGCCGGTGTCGT	GCACTGGCCAGGGGTCTGGT	-	+	+	+
P2Y2.S	GACAACTGGCCCTTCGGCGT	CAAGGCAGCTGTTGGCGCTG	-	+	+	+
P2Y4.L	TGCTGCCGGTCTCCTACAGTGC	TTGATCCCAAGCAACCGGGCA	-	-	-	-
P2Y6.L	ACGTGTGCTCCCTCCCGCTC	ACCAGGCAGGGCACATCGTCC	+	+	+	+
P2Y6.S	CCCGCACGGCCATCTACACC	GACAGGGCCGGAGGGCTCA	+	+	+	+
P2Y8.L	TGGCCTGGGCAGCCTTCATC	GTTCACAGCTGCGATGGGTGC	-	-	-	-
P2Y8.S	AGGCACCTTTGCACGCTGGT	CCTGCCGCTTACAGCTGCGAT	-	-	-	-
P2Y10.L	CACATGGCCGTTCGGACGCT	AAGGCGCCCACGCATCACTG	-	-	-	+
P2Y10.S	ACCGCAAGTGGGCGTTTGGA	TCCGTGCTTCGCGACCTCAT	-	-	-	+
P2Y11.L	GGAGTTTGTGGTGTCTGTGCTCGG	ACAGGGGCAGAGATGGCAGC	-	+	+	+
P2Y11.S	TGGGCCTGGGGGCTCAAAGA	AGCAGCGGGTGGATACAGGGA	-	+	+	+
P2Y12.L	TGGGTTGGCAGTGCGTGTGT	GGCCGGCCTTTTTCTCCACA	+	+	+	+
P2Y12.S	TTCGAAACTCGGGCTATGGAAGC	TCCACATGGCCAGCAGCGAG	-	-	-	-
P2Y13.L	TGGCTCCTCATTGCTCCCCA	AGCAGCCAGCCAAAGCGTGA	-	-	-	-

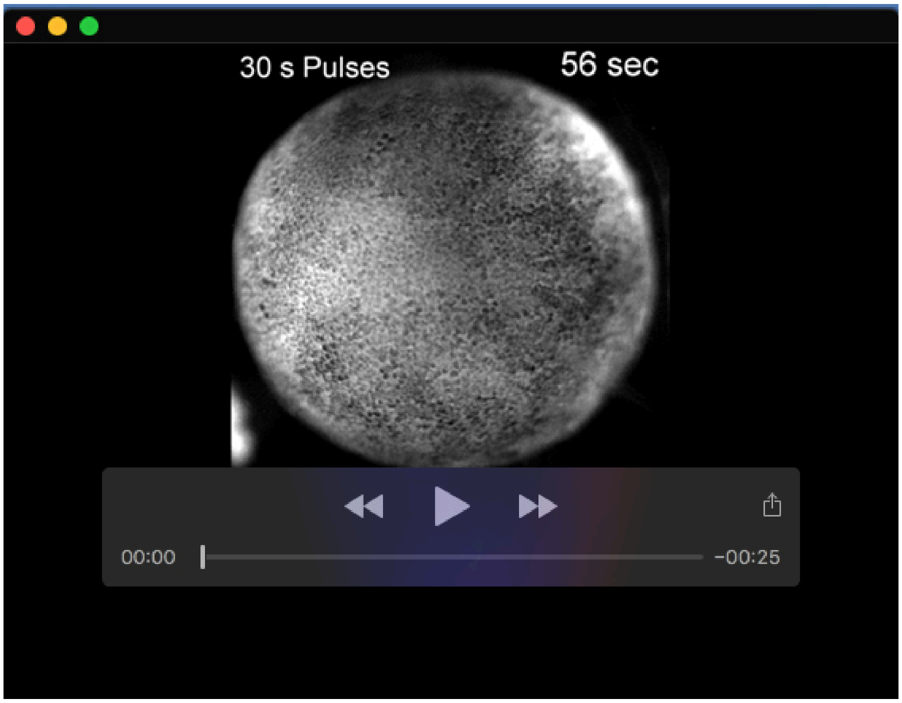

Movie 1. (supporting Fig. 1B). Time-lapse sequence of 10 repeated perfusions delivered at 30 second intervals. Contraction response saturates over the perfusion sequence.

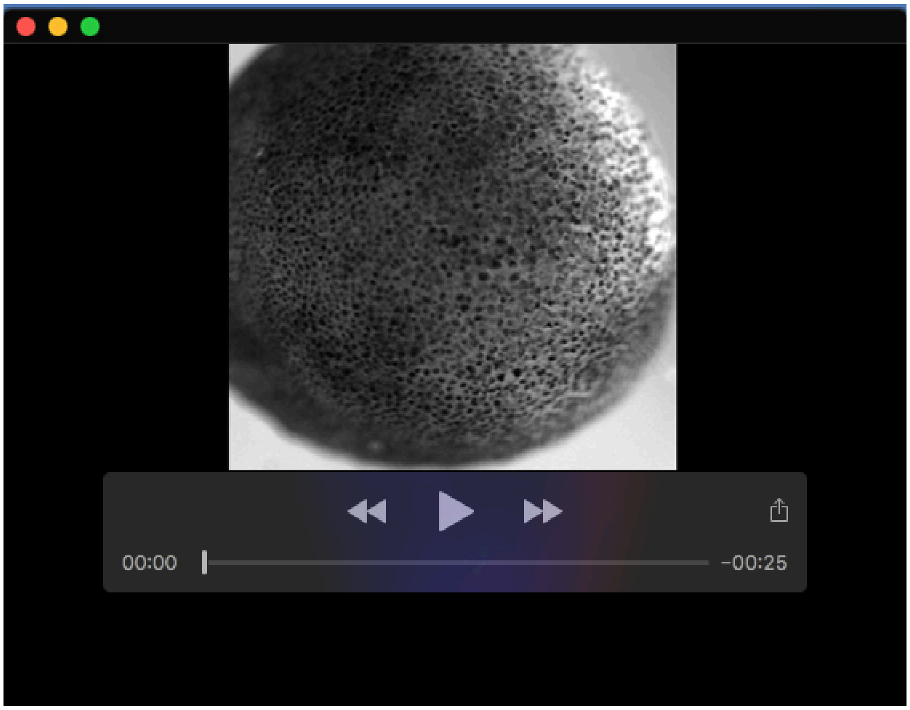

Movie 2. (supporting Fig. 1C). Time-lapse sequence of 9 repeated perfusions delivered at 600 second intervals. Contraction relaxes between perfusions but the response does not desensitize.

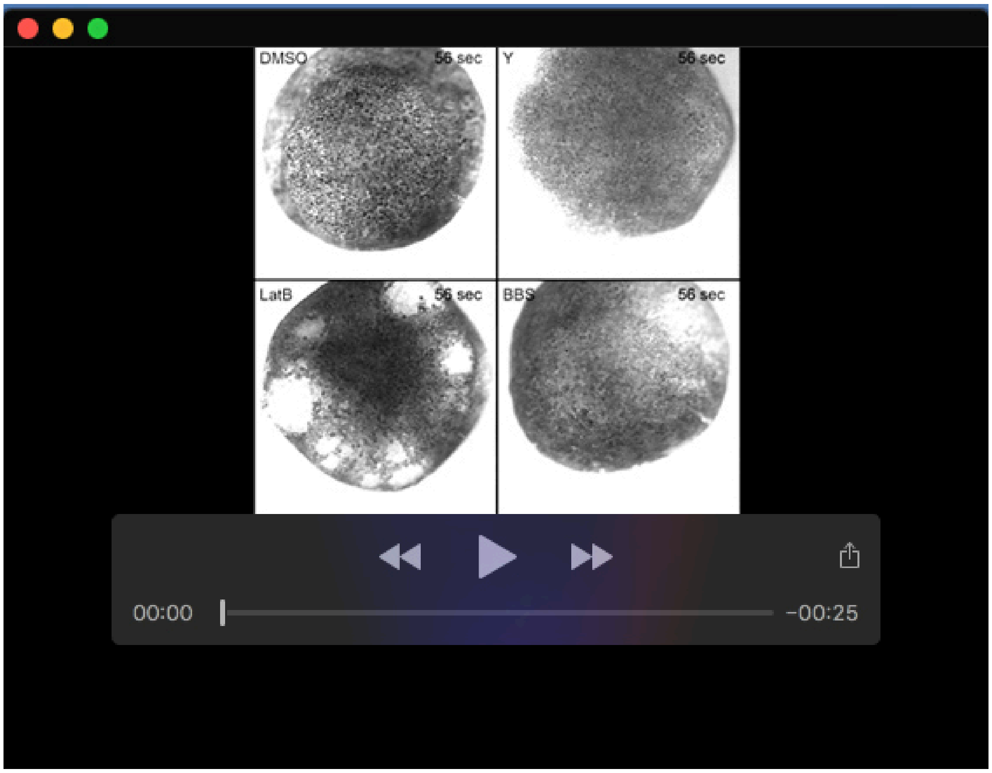

Movie 3. (supporting Fig. 2A). Time-lapse sequences of four embryos incubated in carrier control DMSO (top left), Y27632 (top right), Latrunculin B (bottom left), and Blebbistatin (bottom right).

Journal of Cell Science • Supplementary information
